# The impact and challenges of digital marketing in the health care industry during the digital era and the COVID-19 pandemic

**DOI:** 10.3389/fpubh.2022.969523

**Published:** 2022-07-28

**Authors:** Sahala Benny Pasaribu, Dewiana Novitasari, Francisca Sestri Goestjahjanti, Tonny Hendratono

**Affiliations:** ^1^Faculty of Economics and Business, Trilogi University, South Jakarta, Indonesia; ^2^Economics and Management Department, Sekolah Tinggi Ilmu Ekonomi Insan Pembangunan, Tangerang, Indonesia; ^3^Tourism Department, Sekolah Tinggi Pariwisata Ambarrukmo, Yogjakarta, Indonesia

**Keywords:** digital marketing, health care industries, digital era, COVID-19, pandemic, hospital

## Introduction

According to Arni and Laddha (1), the healthcare industry was under great pressure due to the emergence of COVID-19. The COVID-19 pandemic has had devastating effects on the global economy, industries, and organizations, affecting marketing and spending strategies. Due to the total lockdown in various regions, digital marketing is crucial because traditional marketing strategies are no longer working. Due to a large number of quarantine patients at home, as well as concerns about the possibility of contracting COVID-19, people have chosen not to visit health care facilities, both clinics and hospitals, resulting in a decrease in the volume and income from prescribing drugs from 2021 until now. Khan and Nawaz (2) stated that digital marketing strategies have increased over the years, and spending on such plans has also increased. The COVID-19 pandemic accelerates the growth rate of digital marketing in the health industry and is expected to increase the return of both direct visits and patient telemedicine to hospitals so that hospital revenues also increase.

The development of the Internet, the World Wide Web, and digital technologies such as technology platforms from desktops, laptops, smartphones, to tablet devices used by consumers has changed marketing. Patients, who are convinced that they are consumers of health services and products, are increasingly using the internet or other digital technologies to find the right information, as well as choose and buy those goods or services. For organizations like hospitals, especially during the COVID-19 pandemic conditions, digital media and new technology platforms provide opportunities to expand into new markets, offer new services, apply new online communication techniques, and compete on a more equal footing with larger businesses. According to Arni and Laddha (1) and Al-Weshah et al. (3), most consumers searched for information online about treatment options or more generally, to learn about health problems or health care providers. Digital marketing can simply be defined as achieving marketing goals through the application of technology and digital media. Hospital use of digital technology increased by 50% to reach healthcare consumers. According to de Ruyter et al. (4) and Khan and Nawaz (2), 48% of healthcare provider executives see revenue growth

as a key benefit of digital investment. Today, consumers are looking for a stress-free and trustworthy user experience. With the advancement of technology, the demand for innovative healthcare applications has increased. Therefore, the implementation of health marketing strategies on digital platforms will enable the health sector to grow.

## Discussion and opinion

**Figure 1:**
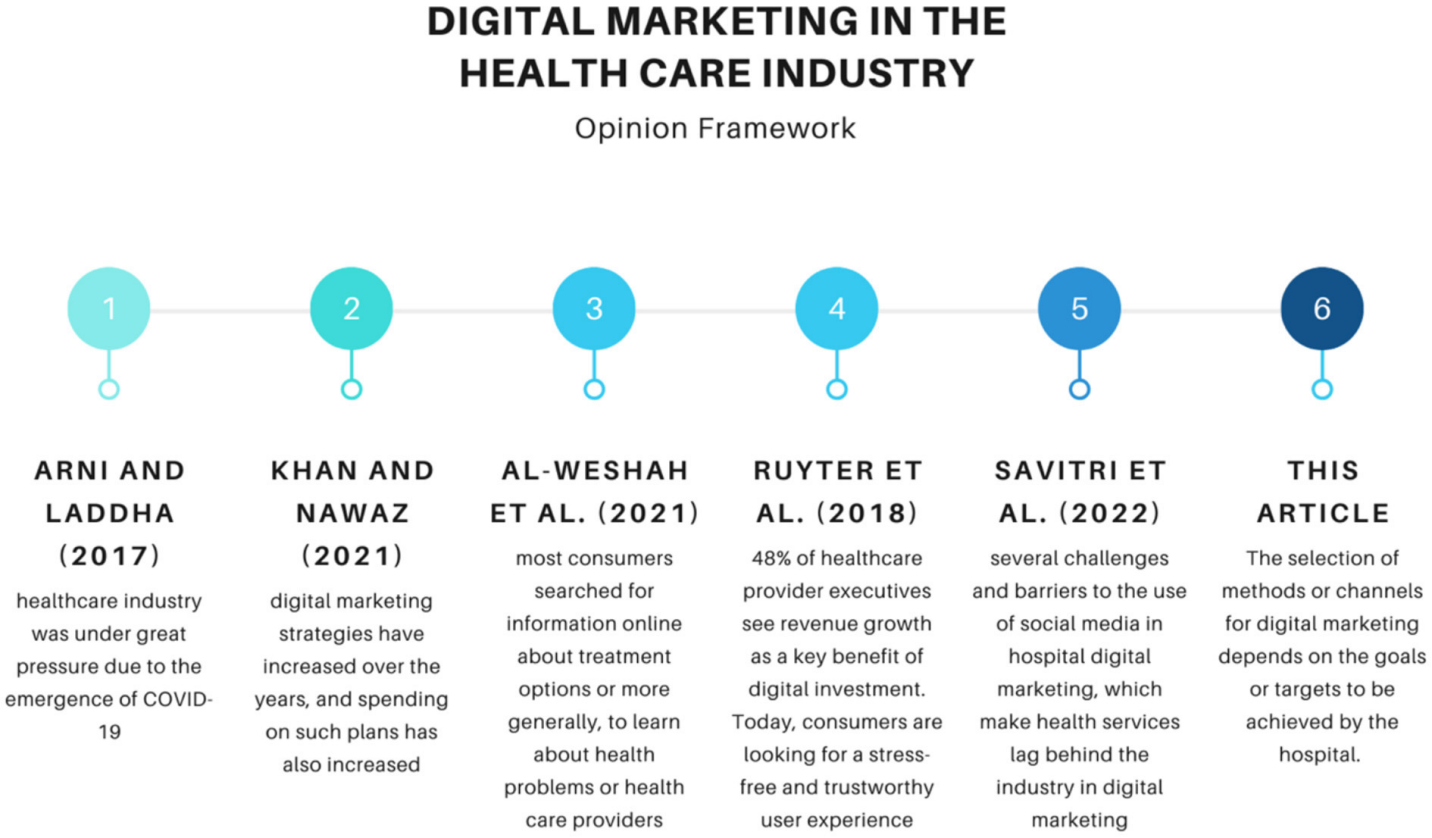
Opinion framework.

Digital methods can promote medical services in order to expand the business. The strategic way of thinking, in this case, implies attracting new patients and offering them quality health services, which ensures satisfaction and the possibility for them to recommend further health facilities. This is consistent with the data from Mishra that digital marketing increases satisfaction, loyalty, and patient engagement with hospital services. According to Wijaya et al. (5) and Wisetsri et al. (6), one of the benefits of digital marketing is to expand the brand/business online. The results of a study in Bangladesh stated that in order to collect information about doctors, people rely on hospital websites because it is the most accurate and up-to-date source of information, and collect information posted on social media such as Facebook groups. Before deciding to visit a health service, people research health care professionals and share reviews about their experiences so that they can be useful for other people in the future. Many people go to hospitals or doctor websites for reviews, and use Google reviews to examine and share medical experiences. In addition, a large number of people use social media platforms to share their experiences. In previous research, we can see in Figure 1 that the opinion framework shows a shift in digital marketing in the current health industry.

Content marketing has a positive impact on factors such as customer engagement, trust, and loyalty. In comparison, content marketing was found to be more effective at persuading customer loyalty compared to trust and loyalty. Furthermore, customer trust in a brand has a strong positive relationship with loyalty. A positive effect of customer engagement on trust has also been found. Digital marketing increases patient satisfaction, loyalty, and engagement with hospital services. According to Savitri et al. (7), Tancharoenwong (8), and Qian et al. (9), one of the benefits of digital marketing is that it can be closer to consumers. Hospitals should adopt a multi-channel content marketing approach to take full advantage.

According to Nunan and Di Domenico (10), Purcarea (11), Purwanto (12), and Pourkarim et al. (13), digital marketing effects and barriers are something to manage during COVID-19. A digital marketing strategy is adopted to manage work online and persist with safety precautions. Technology brings both negative and positive impacts to the society. However, during the pandemic, digital marketing tools and management are still effective and helpful in moving normal life into the new norm. Digital marketing has gained a lot of advantages in terms of online businesses, small, or large entrepreneurs, excelling in the advertising market well.

According to Savitri et al. (7), Tancharoenwong (8), Qian et al. (9), Wijayaa et al. (5), and Wisetsri et al. (6), several challenges and barriers to the use of social media in hospital digital marketing, which make health services lag behind the industry in digital marketing, include security issues, patient privacy, regulatory issues, lack of guidance on how to use digital platforms properly, lack of staff interest to use social media or the right infrastructure to respond to complaints, and unclear responsibilities for various internet marketing activities. As a result, management commitment and consistency are needed because the use of digital technology requires adequate resources in terms of finance, infrastructure, and manpower to be more effective. Electronic communications should be conducted through an experimental approach rather than a planned approach to avoid poor integration of online and offline marketing communications.

## Conclusion

Digital technology marketing has a tremendous impact, including increasing engagement on social media and marketing, serving as a key performance indicator for analyzing organizational values, increasing demand for digital, growing product searches among users, and increasing demand for content platforms. All of them have been demonstrated in many companies and organizations. During the COVID-19 pandemic, digital marketing has hit its growth charts and has made technological advances all over the world. Digital marketing in hospitals during this pandemic is a marketing strategy that has many benefits, including attracting new patients, expanding business, increasing customer/patient trust, strengthening customer/patient loyalty, increasing brand awareness, encouraging patients to use hospital services, and promoting the services to patients' relatives and family. The selection of methods or channels for digital marketing depends on the goals or targets to be achieved by the hospital. After determining the goals of digital marketing, the hospital determines the choice of methods or digital media channels that will be used, then determines the goals, targets, and objectives to be achieved from each of the selected media, analyzes the situation and audience/market share, and determines the budget and marketing frequency. Furthermore, for its implementation, it is necessary to determine who will carry out the marketing strategy that has been chosen. Moreover, monitoring and evaluation of each digital marketing strategy that has been implemented must be carried out so that the hospital can achieve its goals or targets effectively and efficiently.

## Author contributions

All authors listed have made a substantial, direct, and intellectual contribution to the work and approved it for publication.

## Conflict of interest

The authors declare that the research was conducted in the absence of any commercial or financial relationships that could be construed as a potential conflict of interest.

## Publisher's note

All claims expressed in this article are solely those of the authors and do not necessarily represent those of their affiliated organizations, or those of the publisher, the editors and the reviewers. Any product that may be evaluated in this article, or claim that may be made by its manufacturer, is not guaranteed or endorsed by the publisher.
